# Forsythe versus Law

**Published:** 1902-03-08

**Authors:** 


### The Hospital

A Journal of

The Medical Sciences and Hospital Administration.

Vol. XXXI.—No. 806.] Edited by Sir Henry Burdett, K.C.B., and Solomon C. Smith, M.D., M.R.C.P. [March 8, 1902.

### FORSYTHE VERSUS LAW.

The case of Forsythe *versus* Law was one in some respects of so exceptional a character that we are not very likely to witness any reproduction of it, but which, nevertheless, was fraught with lessons to those who will take advantage of them, especially with regard to the unwisdom of permitting friendship or benevolence, in relation to certain classes of patients, to carry a practitioner one step beyond the strictest limits of professional duty. It appeared from the evidence of Eliza Critchlow, who described herself as "a professional nurse," but whose name does not appear in Burdett's Official Nursing Directory, that she nursed the plaintiff at Bath about ten years ago, that she was then suffering from "hysteria and asthma," and was under the care of Dr. Kerr. The witness and the plaintiff met again in 1896, when the latter was "much better," and no more until January 1899, when the plaintiff was staying at Pelham Crescent, and Dr. Law (the defendant) was attending her. He used to call, said the witness, "once or twice every day, and stayed two or three hours. Miss Forsythe had morphia injections by Dr. Law's orders. Sometimes Dr. Law administered the morphia himself."

At this point of the case, the witness appears to have definitely exceeded what is commonly regarded as the province of the nurse, and to have encroached upon that of the physician; for she told Dr. Law that the morphia was doing the patient harm, and then, "in consequence of what she saw, thought it her duty to communicate with the patient's family." Soon after this, a Dr. Kirkland appears upon the scene, the plaintiff during the interval having been treated by Dr. Hardwick, of Hampstead, who diminished the doses of morphia, after which the plaintiff "went away," doing so, in Dr. Hardwick's opinion, because she could no longer obtain the amount of morphia she wished for. Either immediately or soon after she was placed in a nursing home at Ladbroke Grove under Dr. Kirkland, who entirely deprived her of morphia, and who admitted in his cross-examination that she did "come very near to death" in consequence. The general tenor of his evidence was strongly condemnatory of Dr. Law's practice; and he was present at an interview between Dr. Law and the plaintiff's brother-in-law, accompanied by his solicitor, at which suggestions as to "compensation" by Dr. Law were thrown out. The general impression left by this part of the case was that the opinions held by Dr. Kirkland, and his method of expressing them, were in all probability among the determining causes of the litigation which ensued.

In reply to the various allegations, the case of the defendant was a very simple one. He had formerly been in practice in Norfolk Crescent, and the plaintiff had been introduced to him as a nurse. He had employed her in that capacity, had become acquainted with her family, and when, in partnership with a Miss Ward, she opened a nursing home, he had visited both ladies as a friend, and had fallen into the habit of prescribing gratuitously for any ailments from which they might be suffering. In 1895 he sold his practice, and went to live at Ripley, where his wife had property; but he was often in town, and often visited Miss Forsythe and her partner. The conditions of sale of his practice had presumably been such as not to preclude him from gratuitous attendance upon friends, and he continued to prescribe for the plaintiff, and to visit her at the various places to which she went when she was no longer well enough to carry on the management of the home. According to his view of the case, she suffered from spasmodic asthma of a very severe and obstinate character, and obtained from injections of morphia an amount of relief which could not otherwise be afforded her. He brought other physicians from time to time to see her in consultation, and they concurred with him in the treatment. Knowing her means to be very slender, he procured medicines for her at a reduced price, and, among other devices for diminishing the quantity of morphia which she took, he himself diluted the solutions before leaving the bottles at her lodgings.

After hearing Dr. Law's evidence in chief and his cross-examination, the jury said that they were satisfied, and, although Sir Edward Clarke insisted upon addressing them on behalf of the plaintiff, they not only returned an immediate verdict for the defendant, but also characterised the action as one which should not have been brought—an expression of opinion in which the Judge agreed with them, and which will, we think, be fully endorsed alike by the profession and by the public. It is none the less manifest that the very possibility of the proceedings rested from first to last upon a lamentable absence of discretion. If Dr. Law had abstained from mixing together the characters of friend and physician, had paid only necessary visits of only necessary duration, and had committed the morphia syringe to safe hands, such as those of Miss Ward or of any other trained nurse, with explicit instructions as to dosage and repetition, no trouble of any kind would have arisen. In the same way, if Dr. Kirkland had confined himself to doing what he thought best for his patient, and had refrained from expressing opinions as to the course which had been pursued before he saw her, it would hardly have been possible for her or her family to take the case into court. There are certain principles of medical ethics, and certain forms of professional prudence, which cannot be departed from without grave risk to all concerned.

